# Supplementary material for: The Paternal Experience of Fear of Childbirth: An Integrative Review
Source: Int J Environ Res Public Health. 2021 Jan 29;18(3):1231. doi: 10.3390/ijerph18031231 (PMC7908162; doi:10.3390/ijerph18031231)
Supplement: Supplementary file 1 [file ijerph-18-01231-s001.pdf]

Table S1: Thematic synthesis table

Study	Coding	Findings
Eriksson et al. (2007) [40]	Content of fears	The content of the fear was primarily described as being related to the health and life of their partner and child. The main worry concerned the woman. Another concern was related to the obstetrical staff's competence and behaviour. For several men the fear was connected to their own capabilities in a situation of strain. Doubts about their own reactions were common. Some of the men said they had been afraid because the woman was afraid. A few men expressed concerns about their future sexuality. One man feared he might realize that he couldn't be the father of the child, another had feared having a second daughter.<br>*Manifestation:*
	Mental health symptoms	Often described as a mental occupation. Some men said that they had thought about their fear to a very great extent. Others said that that their fear had not been that consuming but still brought with it an increased vigilance. One main described being constantly on the alert and
	Physical symptoms	prepared for the worst. Fear also manifested itself through bodily sensations including reports of uneasiness, restlessness, stomach ache and erectile problems.
	Coping mechanisms	*Ways of dealing with fear:*<br>Increasing their sense of control-<br>Relieving the woman from daily chores to avoid too much pressure
	Medicalisation of birth	being put on her. Restricting the woman from pursuing activities that were perceived as harmful. Initiating more medical attention. Promoting the prospect of having an elective caesarean section. Being continuously prepared for possible events by making different practical
	Avoidance	arrangements. Trying to be prepared for how to behave during childbirth.<br>Diminishing the very emotion of fear-<br>Some men had tried to accomplish this by keeping themselves occupied with other activities such as increasing their rate of work or physical training. A father of two related that he went to the pub the night before labour was planned to be induced. Others withdrew from situations from which childbirth might be discussed. Visiting the antenatal clinic might also be stressful "Having to sit there with the midwife and talk about everything that could happen was not a situation I wasn't really up for, so ultimately I didn't go along that often".
	Non-disclosure	*Reasons for keeping fear to oneself*<br>Most had not explicitly expressed or spoken about their fears to their partner, friends or relatives. The main reason for keeping quiet was out of thoughtfulness for one's partner. Some men said that talking about it

	Gender constructs<br><br>Poor antenatal support	might generate fear in the woman as well. They also did not want to be a burden, disappoint or give the woman the impression that she could not expect support. "I believe in that situation she wanted me to be strong, and if I had said that I was afraid, she would no doubt have felt abandoned". Some men had remained silent since they had the perception that men's fear in this situation was of no interest to anybody. Others argued that talking about their fear would not have made it better, or said it may have made things worse. All men emphasized that talking about fear of childbirth was difficult in some way. Some felt talking about fears was not in the nature of men whereas others talked about societal expectations. "As a man, you're expected to be strong and support your woman, and in that situation it doesn't really seem appropriate to start talking about your own fears". When reflecting on of revealing their fear, some of the men now felt that talking about the fear might have been beneficial and even desirable. One participant had actually tried to receive support in antenatal care but without success. "I noted that there were no routines for dealing with the man's fears".
Hunter et al. (2011) [16]	Religious practice<br><br>Mantram practice	The fathers average W-DEQ score was 87.8 (range 73-96). There was no statistically significant difference for fear between the intervention and control group. There was an inverse relationship between the frequency of religious practice and the W-DEQ fear scale at baseline ($r = -.3$, $p < .10$), indicating that more frequent religious practice was associated with lower fear levels. This inverse relationship was however not maintained post-delivery. Eight participants completed satisfaction questionnaire evaluating the mantram intervention (n=5 mothers, n=3 fathers) six (75%) reported high satisfaction, and two (25%) reported medium satisfaction. None of the fathers completed the six month follow up interview.
Hildingsson et al. (2014b) [43]	Physical symptoms<br><br>Mental health symptoms<br><br>Previous labour experience	Childbirth related fear was present in 13.6% of fathers as assessed using the FOBS with results >50 indicating greater fear. These fathers were more likely to rate their physical (OR 1.8; CI 95% 1.2-2.8) and mental health (OR 3.0; 1.8-5.1) as poor compared to fathers without FOB. Fearful fathers were more likely to perceive difficulties in pregnancy (OR 2.1; 1.4- 3.0) forthcoming birth (OR 4.3; 2.9-6.3) and parenthood (OR 1.4; 0.9-2.0) then fathers without FOB. Higher levels of self-rated stress were also present in men with FOB at 12 months postpartum.<br>Fathers with previous children and child birth fear attended antenatal visits to the midwife more often than fathers without fear (OR 1.1; 95% CI 1.01-1.32) and there was a tendency that their baby's birth was performed instrumentally (OR 3.0; 1.0-8.9).

Eriksson et al. (2006) [4]	Content of fears<br><br>Previous labour experience	Concerns for the health and life of the baby was the most frequently expressed fear by men (79%). Having a diseased or handicapped child (54%), fear of losing the child (12%).<br>The health and life of the woman was the second largest category in men (49%). With (45%) of the men having feared that their partner would be injured. (24%) of men had fears relating to their own capabilities and reactions, (17%) not being able to give help and support, not being able to endure the situation (7%).<br>Professionals competence and behaviour was raised in (5%) of the men. Fears related to the health and life of the baby (P = .004) and the health and life of the woman (P = .003) were described significantly more often by men with intense fear than by men with mild to moderate fear. Fear of prolonged birth and fear of interventions were described more frequently by men with intense fear. Among the parents with more than 1 child, 21% had at least 1 earlier experience of induced labour, 11% vacuum extraction, and 9% emergency and 8% elective caesarean delivery.
Bergström et al. (2013) [13]	Mental health symptoms<br><br>Intervention<br><br>Partners<br><br>Labour outcomes	Men with fear of childbirth more often experienced childbirth as frightening (OR 4.68, 95% CI) and feeling unprepared for childbirth (OR 4.04; 95% CI 2.08-7.84) compared with men without fear. Psychoprophylaxis group had lower risk of experiencing childbirth as frightening compared with those in standard care (OR 0.30: 95% CI 0.10-0.95). (92%) of the men with Fear of childbirth were first time fathers. More men with FOB also had partners suffering from the same condition. No statistical difference in the course of the partners labour was found between men with or without FOB or between intervention group and non-intervention group.
Szeverényi et al. (1998) [44]	Content of fears	Approximately 80% of both the men and women had fears relating to childbirth. 13% of men had a strong fear and 11% a very strong fear of caesarean delivery. 15.7% of men were very afraid that their wife could die and 5.6% quite afraid. 14.8% were very afraid their baby may be stillborn and 11.1% quite afraid. 15 % of men were fearful of doing something wrong during labour.
Hildingsson (2014a) [14]	Desired outcomes<br><br>Labour outcomes	5% of men reported childbirth related fear. Men had strongest attitudes about a birth being the safest and less stressful option for the woman, less painful and a birth that would facilitate a quick recovery. There were no differences between men with childbirth fear and those without in any of the attitudes. Birth preference and fear was strongly associated with mode of birth.
Kannenberg et al. (2016) [46]	Timing	Expectant fathers state anxiety fell significantly with increasing number of children (p=0005). However there was significant increase in trait anxiety (p=0.044) and state anxiety (p=0.016) as pregnancy progressed. Thus men had significantly more pregnancy related anxiety at the end

	Age<br>Parity<br>Content of fears<br>Education	of pregnancy than at the beginning or mid-way through. Anxiety surrounding the birth decreased with increasing age (p= 0.008). Anxiety around the birth did not decrease with parity among expectant fathers as it did with the mothers, the birth of each child is seen as an equally extraordinary event. Anxiety rose as gestational age increased. The most significant anxiety among expectant parents is fear for the unborn child's health, followed by anxiety surrounding the birth/delivery. Fear of foetal malformation are experienced as more anxiety-provoking by parents with higher levels of education than those with lower education. There was no differences between education level groups regarding fears surrounding the birth itself, this event is perceived as equally daunting by all expectant parents.
Hildingsson et al. (2014c) [45]	Demographics<br>Mental health symptoms<br>Nature of pregnancy<br>Medicalisation of birth	13.6 % of fathers (n= 142) scored 50 or more on the FOBS and were included in the 'fear of birth group'. Fathers reporting FOB were more likely born in a country outside Sweden (p= 0.002), as were fathers expecting their first baby (OR 1.8; 1.2-2.6). Fathers with FOB had less positive feelings about the pregnancy and less positive feelings when thinking about the approaching birth, but no such differences were found when investigating their feelings when thinking about the first week with the new born baby. There were no differences in fear scores related to the planned or unplanned nature of the pregnancy or preceded infertility or assisted conception. Fathers with FOB were twice as likely to report that they were thinking about the birth of their baby(OR 1.9; 1.1-2.0).. Men with FOB were also less likely to agree with the statement that giving birth is a natural process that should not be interrupted which is mirrored in the higher preference for CS for the birth of their baby.
Eriksson et al. (2005) [11]	Age<br>Timing<br>Non-disclosure<br>Isolation<br>Content of fears<br>Mental health	13% of the male participants had intense fear of childbirth. Men with intense fear were more often 40 years of age or older than men with mild/moderate fear, and had more frequently more than 5 years elapsed between the first and second child. 56% of men with intense fear said nothing about their fear as they did not want to worry their partner. 49% with intense fear felt it best to keep the fear to myself. 65% of men with intense fear said nobody asked how I felt about the childbirth. 98% of men with intense fear and 92% with mild/moderate fear felt afraid that the child would not be healthy and fine. 86% of men with intense fear felt that in facing childbirth you are expected to be positive and expectant.
Etheridge and Slade (2017) [9]	Content of fears	The experience of being present at birth was one of constant shift and change both in terms of situational aspects and emotional content. The suddenness and speed of situational changes created the impression of the birth as a "rollercoaster", creating a level of uncertainty, anxiety and helplessness. 10 of the 11 men described fears that their partner or baby

	Mental health symptoms<br>Gender constructs<br>Non-disclosure<br>Giving support<br>Receiving support<br>Isolation<br>Physical symptoms<br>Avoidance<br>Flashbacks/dreams<br>Non-disclosure<br>Labour outcomes	would die. The pain of the woman and her suffering had a direct effect on the man and his distress mirrored hers. 7 fathers referred to "trying to keep it together" and be strong for their partner, this seemed motivated by the belief that, for the woman, seeing her partner upset was "not going to do her any good at all". Most of the men tried to hide their feelings but some were so overwhelmed they broke down. Others were able to contain their emotion during the birth but became extremely distressed when on their own and "just broke down in tears". A feeling of utter helplessness was a key theme with men perceiving themselves unable to do anything practical, alongside feelings of uncertainty from a lack of information and understanding. The men put their trust in the health professionals which gave them a sense of unease. Isolation during the birth increased anxiety and uncertainty about what was happening, intensifying the men's fears that something went wrong. Several men reported changes in perception and a "heightened awareness" as the events of the birth took place. Preoccupation and rumination was a feature for some men in the weeks, months and even years after the birth. External stimuli, such as television programmes of babies being born, triggered memories of the birth and as a consequence they tried to avoid them. Some men had flashbacks to the birth or relived it in their dreams. The expectation that others may not understand or would dismiss their distress also affected how fathers shared their experiences of birth. Fathers indicated that they did not feel that they had the right to be affected because "nothing actually happened to me". All participants described births in which complications had arisen.
Schytt and Hildingsson (2011) [48]	Mental health	Poor emotional self-rated health was associated with having children previously, childbirth related fear, pronounced emotional changes during pregnancy and perceived stress when facing the forthcoming parenthood. . 30 % of those with childbirth related fears rated poor physical self-rated health and 27% poor emotional self-rated health in late pregnancy. With poor physical self-rated health among 42% and poor emotional self-rated health in 37% one year after birth in those with childbirth fear.
Somers-Smith (1998) [42]	Content of fears<br>Physical symptoms<br>Mental health symptoms<br>Giving support<br>Non-disclosure	One fear voiced by two men (25%) was the possibility of their partner dying. Other fears the men expressed were the possibility of fainting, panicking and if they would be able 'to keep it together'. Two fathers were concerned that they would fail to meet the needs of their partner. The men mostly kept their fears to themselves. The men wanted to be

	Receiving support	of practical help to their partner. One father relied on cues from the midwife to minimise his anxiety during the labour.
Greer et al. (2014) [41]	Perception of risk<br><br>Content of fears<br><br>Mental health<br><br>Medicalisation of birth<br><br>Giving support<br><br>Receiving support	Over half of the men (n=9) appraised labour and vaginal delivery as posing considerable risks to the physical health of the mother and baby. 42% of the men feared their baby was too big to be born vaginally. 74% (n= 14) of the men were fearful that their partner would be unable to cope with, and would be traumatised by the pain. The men feared that being traumatised by the birth would affect their partner's postnatal mental health and impact negatively on the new family unit. All of the men and women who expressed opinion about CS said it was a safer mode of delivery for the baby. Perception of riskiness of vaginal birth was increased when there were previous negative birth experiences. 58% of men feared they would be unable to provide adequate labour support. 4 men expressed more confidence in their ability to provide practical help following a CS than support during active labour. All men wanted their partner to have as much pain relief as possible during the birth. The men were more positive about the use of medical interventions in the birth than were the women. Two multiparous women and their partners identified midwife support as a resource to help them cope with birth, recalling how the midwife had reduced their fears.
Schytt and Bergström (2014) [17]	Age<br>Mental health symptoms<br><br>Labour outcomes	Expectant first-time fathers' feelings and expectations of the upcoming birth varied by age. The older men had more negative feelings than the younger ones. 29% of the advanced aged men had had mixed or negative feelings compared with 27% average age and 17% young age (p=<0.05). Fearful expectations were found most among the oldest. The total sum score on the W-DEQ for men in the oldest age category was 43.3 (SD 16.9), compared with 42.9 (SD 13.5) in men of average age and 38.7 (SD 15.7) in the youngest. The older men assessed their partner's labour and birth as more difficult (advanced age 42%, average age 41% and young 32%; p= 0.05).
Chalmers and Meyer (1996) [47]	Content of fears<br><br>Mental health symptoms<br><br>Receiving support	The most significant fears experienced by the men during the pregnancy was the fear of abnormality in the baby (71%), 50% reported checking the baby for abnormality at the birth, not being at the delivery (47.8%), partner experiencing pain (43.5%), partner or baby dying (41.3%). 30.4% of men reported feeling more anxious than before pregnancy, more emotional (13.4%) and more irritable (8.7%). The most important source of emotional support for men was their partner (63.9%).
Shibli-Kometiani and Brown (2012) [1]	Mental health symptoms<br>Content of fears	Every participant expressed significant levels of anxiety, fear and helplessness as labour progressed. As their distress increased they became passive and less supportive. They were concerned that their

	Giving support<br><br><br>Receiving support	partners and babies might die. Most found that giving support was harder than expected. Fathers were not only surprised by their levels of discomfort but were dissatisfied with their own performance on contrast with their earlier beliefs of their ability to support their partners. Most of the men felt the need for support themselves. Knowledge deficit about labour served to increase their anxiety.
